# Frequency of Subclinical Atherosclerosis in Brazilian HIV-Infected Patients

**DOI:** 10.5935/abc.20180058

**Published:** 2018-05

**Authors:** Péricles Sidnei Salmazo, Silméia Garcia Zanati Bazan, Flávio Gobbis Shiraishi, Rodrigo Bazan, Katashi Okoshi, João Carlos Hueb

**Affiliations:** Faculdade de Medicina de Botucatu (UNESP), Botucatu, SP - Brazil

**Keywords:** Atherosclerosis / complications, HIV, Cardiovascular Diseases / mortality, Carotid Intima Media Carotideo, Vascular Stiffness, Risk Factors

## Abstract

**Background:**

AIDS as well as atherosclerosis are important public health problems. The longer survival among HIV-infected is associated with increased number of cardiovascular events in this population, and this association is not fully understood.

**Objectives:**

To identify the frequency of subclinical atherosclerosis in HIV-infected patients compared to control subjects; to analyze associations between atherosclerosis and clinical and laboratory variables, cardiovascular risk factors, and the Framingham coronary heart disease risk score (FCRS).

**Methods:**

Prospective cross-sectional case-control study assessing the presence of subclinical atherosclerosis in 264 HIV-infected patients and 279 controls. Clinical evaluation included ultrasound examination of the carotid arteries, arterial stiffness by pulse wave velocity (PWV) and augmentation index (AIx), laboratory analysis of peripheral blood, and cardiovascular risk according to FCRS criteria. The significance level adopted in the statistical analysis was p < 0.05.

**Results:**

Plaques were found in 37% of the HIV group and 4% of controls (p < 0.001). Furthermore, carotid intima-media thickness was higher in the HIV group than in controls (p < 0.001). Patients with carotid plaque had higher fasting glucose, total cholesterol, low-density lipoprotein cholesterol, and triglycerides than those without plaques. The presence of HIV, adjusted for age, overweight/obesity, and smoking increased by almost fivefold the risk of atherosclerotic carotid plaque (OR: 4.9; 95%CI: 2.5-9.9; p < 0.001). Exposure to protease inhibitors did not influence carotid intima-media thickness, was not associated with carotid plaque frequency, and did not alter the mechanical characteristics of the arterial system (PWV and AIx).

**Conclusions:**

HIV-infected patients are at increased risk of atherosclerosis in association with classical cardiovascular risk factors. Treatment with protease inhibitors does not promote functional changes in the arteries, and shows no association with increased frequency of atherosclerotic plaques in carotid arteries. The FCRS may be inappropriate for this population.

## Introduction

By the end of 2012, about 35 million people were HIV positive worldwide. By June 2012 in Brazil, 656,701 cases had been identified since the first one detected in São Paulo in 1980; this includes 253,706 lethal cases between 1980 and 2011.^1,2^ In the mid-1990s rates were increasing, but the current situation indicates a stable epidemic,^2^ with signs of a reduction in mortality rate in the last decade.^1^ The most important contributing factors were the introduction and easy access to highly active antiretroviral therapy (HAART). However, over the years, observations have shown that HAART may alter the patient's lipid profile, thereby accelerating atherosclerosis.^3-8^ Despite this, cardiovascular disease (CVD) is the leading cause of death worldwide (World Health Organization, 2013) and represents the foremost cause of preventable death.

There is some indication of a possible direct effect from viral protein particles or infected cells liberating proteins in the receptors present in the vascular endothelium, thus favoring the presence of pro-coagulants, platelet activation, reduced nitric oxide production from the destruction of CD4 T lymphocytes (CD4^+^ cell), and the production of inflammatory cytokines.^4,9,10^ Recent publications have indicated that viral effect on the vascular endothelium can contribute to a reduction in the number of primary endothelial cells, which leads to endothelial dysfunction and atherosclerosis.^11^

Nevertheless, there is no consensus on the relationship between HAART and atherosclerosis; this may be due to the complexity of the factors involved.^4-6,8,10,11^ In light of these facts, new strategies have been suggested to prevent cardiovascular events, including subclinical atherosclerosis research.^6,12-16^

Carotid intima-media thickness rate (CIMT) and the presence of atherosclerotic plaque (PL) in the carotid have been associated with the Framingham coronary heart disease risk score (FCRS); individuals with this index elevated have a higher risk of developing CVD.^17-24^ Another marker of CVD is high-sensitivity C-reactive protein (hs-CRP). In HIV-positive patients, hs-CRP, although low in sensitivity, is known to be a possible marker of disease progression and atherosclerosis.^25-28^

Arterial stiffness by pulse wave velocity (PWV), augmentation index (AIx), and ascending aortic pressure (AP) have been studied as promising indices of early endothelial dysfunction diagnosis.^29-32^ Few publications have evaluated these indices in HIV-positive patients and the number of cases has been limited.^16,29-32^

The objectives of the present study were: 1- To identify the frequency of subclinical atherosclerosis in HIV-positive patients, comparing it with that of control subjects; 2- To associate the diagnosis of subclinical atherosclerosis with viral load, CD4 levels and antiretroviral treatment in HIV-positive patients; 3- To associate the presence of carotid atherosclerosis with cardiovascular risk factors and with the FCRS in HIV-positive patients.

## Methods

Written informed consent was obtained from all participants, and the study protocol was approved by the Ethics Committee of the university.

This is a prospective cross-sectional case-control study with consecutively selected patients.

All HIV-infected patients from the Infectious Diseases outpatient clinic were included in the study. Exclusion criteria were evidence of atherosclerosis (interview, chart review and physical examination), age under 18 years, pregnancy, evidence of other causes of immunosuppression, and data acquisition failure due to technical difficulties.

Healthy controls were prospectively included.

### Data source

Invitation to participate in the study was offered after exposure to the project in the waiting room during a routine visit.

Those who accepted were referred to a clinic where they received more information, had their doubts clarified, and underwent an interview guided by a structured questionnaire, a physical examination, and a carotid ultrasound assessment followed by a referral to collect a blood sample for laboratory tests.

HAART information, time since diagnosis and treatment, HIV-RNA viral load, and CD4^+^ and CD8^+^ cell counts were obtained from a review of medical records. Cardiovascular risk was calculated by FCRS.^12^

#### Carotid artery ultrasound

Carotid artery ultrasound was performed by the same appropriately trained expert using a Vivid I or Vivid S6 (General Electric Healthcare, USA) equipped with 7.0 MHz linear transducer and an image acquisition system. Images were obtained and analyzed according to the Consensus Statement from the American Society of Echocardiography and Mannheim Carotid Intima-Media Thickness Consensus recommendations.^21,22^

Carotid intima-media images were obtained by an automated method using GE developed software, to determine average thickness of the left and right carotid arteries.

A PL was defined as a focal structure that encroached into the arterial lumen at least 0.5 mm, or 50% of surrounding CIMT, or carotid thickness > 1.5 mm.^21^

#### Arterial stiffness

Arterial stiffness indices (PWV, AIx, and AP) were obtained by the same experienced operator using Sphygmocor CPV System equipment (AtCor Medical, Australia) and following current recommendations.^29^

#### Laboratory tests

A sample of 12-hour fasting peripheral blood was obtained from all patients to analyze hs-CRP, glucose, albumin, complete blood count, urea, creatinine, total cholesterol (TC), high-density lipoprotein-cholesterol (HDL-c), and triglycerides (TGL). LDL-c was estimated using the Friedewald equation when TGL were lower than 400 mg/dL.^7^

### Statistical analyses

All statistical analysis was performed using SAS/STAT (SAS Institute Inc., Cary, North Carolina, USA).

Continuous variables with normal distribution were presented as mean and standard deviation, and continuous variables with non-normal distribution were presented as medians and interquartile ranges. Categorical variables were presented as proportions. The Shapiro-Wilk test was performed as a normality test.

Multivariate logistic regression was used to estimate associations between carotid atherosclerosis and clinical variables.

Multiple linear regression was utilized to analyze associations between arterial stiffness and clinical variables or the presence of carotid atherosclerosis.

The Wilcoxon-Mann-Whitney test was used to compare two groups of non-parametric results. The unpaired Student *t* test was applied for parametric results.

One-way ANOVA was employed to compare the groups in FCRS classification.

All tests were two-tailed, and significance was set at p < 0.05.

## Results

### Study population

The study included 264 HIV-infected patients and 279 healthy volunteers (control group). In the HIV-infected group, median time since HIV diagnosis was 96 months (35-149 months) and treatment duration was 78 months (15-142 months). Viral load ranged from undetectable to 397,155 copies/mL (median: undetectable; 75^th^ percentile: 253 copies/mL). CD4^+^ counts ranged from 442 to 16,338 cells/µL (median: 1,739 cells; interquartile range: 1,350-2,212 cells). Of the HIV-infected patients, 35 were without HAART.

Table 1 shows the demographic and clinical variables of the HIV-infected patients and controls. Compared to controls, HIV-infected patients were six years older (43.2 ± 10.5 vs. 37.9 ± 11.5 years; p < 0.001), had lower BMI (25.5 ± 4.5 vs. 27.4 ± 5.4 kg/m^2^; p < 0.001), lower frequency of overweight/obesity (51.1 vs. 63.1%; p = 0.005), and higher active smoking incidence (43.6 vs. 16.1%; p < 0.001).

**Table 1:** Demographic and clinical variables of HIV-infected patients and the control group.

Variables	HIV group (n = 264)	Control group (n = 279)	p
Age (years)	43.2 ± 10.5	37.9 ± 11.5	< 0.001
Sex (F/M)	125/139	144/135	0.321
O_ob (yes/no)	135 (51,1%)/129	176 (63.1%)/103	0.005
SAH (yes/no)	28/236	23/256	0.360
Smoking (yes/no)	115 (43.6%)/149	45(16.1%)/234	< 0.001
Diabetes (yes/no)	10/254	6/273	0.263
BMI (kg/m^2^)	25.5 ± 4.5	27.4 ± 5.4	< 0.001
SBP (mm Hg)	121 (111;133)	120 (110;130)	0.535
DBP (mm Hg)	77 (71;85)	80 (70;80)	0.616

F: female; M: male; O_ob: overweight/obesity; SAH: systemic arterial hypertension; BMI: body mass index; SBP: systolic blood pressure; DBP: diastolic blood pressure.

Table 2 shows clinical and laboratory variables of the patients, separated in treatment with protease inhibitors (PI). Those exposed to PI showed longer time since diagnosis [140 (74-175) vs. 72.5 (20-120) months; p < 0.001] and disease treatment duration [124 (56-155) vs. 44 (4-101) months; p < 0.001] and elevated TGL levels [190 (119-280) vs. 140 (100-188.5) mg/dL; p < 0.001]; but PI exposure had no effect on LDL-c, HDL-c, fasting glucose, creatinine, or hs-CRP levels.

**Table 2:** Clinical and laboratory variables of the patients treated or not with protease inhibitors

Variables	PI + (n=116)	PI - (n=148)	p
Time since diagnosis (months)	140 (74;175)	72.5 (20;120)	<0.001
Disease treatment duration (months)	124 (56;155)	44 (4;101)	<0.001
LDL-c (mg/dL)	103.2 (80.8;132.4)	102 (83.4;132.8)	0.796
HDL-c (mg/dL)	42 (35;56)	45 (37;53)	0.626
TGL (mg/dL)	190 (119;280)	140 (100;188.5)	<0.001
Fasting glucose (mg/dL)	83 (77;91)	83 (77;94)	0.764
Creatinine (mg/dL)	0.80 (0.70;1.0)	0.80 (0.70;0.90)	0.067
Hs-CRP (mg/dL)	0.50 (0.30;0.70)	0.50 (0.30;0.80)	0.344

PI +: in use of protease inhibitors; PI -: no use of protease inhibitors; LDL-c: low-density lipoprotein; HDL-c: high-density lipoprotein; TGL: triglycerides; hs‑CRP: high‑sensitivity C reactive protein.

### Atherosclerotic plaques in carotid arteries and carotid intima-media thickness

**Figure 1:**
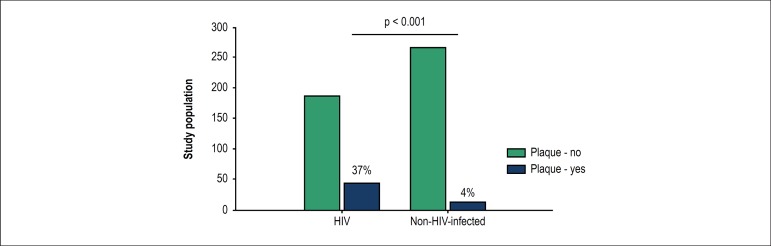
Frequency of carotid artery plaque in HIV-infected patients and non-HIV-infected controls.

Plaques were detected in 37% of the HIV group and 4% of the control group (p < 0.001), as shown in Figure 1.

Multivariate logistic regression analysis indicated that the presence of HIV, adjusted for age, overweight/obesity, and smoking, had an almost five-fold increase in the risk of carotid PL (OR 4.9, 95% CI 2.5 to 9.8; p < 0.001).

Patients with PL were 11 years older than those without PL (51.4 ± 9.21 vs. 40.2 ± 9.40 years, p < 0.001), and had higher levels of fasting blood glucose [90 (78-100) vs. 83 (76.5-90) mg/dL; p = 0.012], TC [200 (178-244) vs. 181 (156-208.5) mg/dL; p < 0.001], LDL-c [120.1 (96.2-148.4) vs. 96.8 (80-125) mg/dL; p < 0.001], TGL [188.5 (125.5-288.5) vs. 150.5 (108-226) mg/dL; p = 0.010] and creatinine [0.80 (0.70-1.10) vs. 0.80 (0.70-0.90) mg/dL; p = 0.027].

Patients with PL also had higher systolic (SBP: 132 ± 21 vs. 121 ± 16 mm Hg; p < 0.001) and diastolic blood pressure (DBP: 83 ± 12 vs. 77 ± 11 mm Hg, p < 0.001). In addition, PL was detected in approximately 34% of men versus 17.4% of women.

Regarding treatment with PI, exposure to this drug class was not associated with higher PL frequency. Nevertheless, results show significant interaction between PI and elevated TGL, even though no association with the presence of PL.

**Figure 2:**
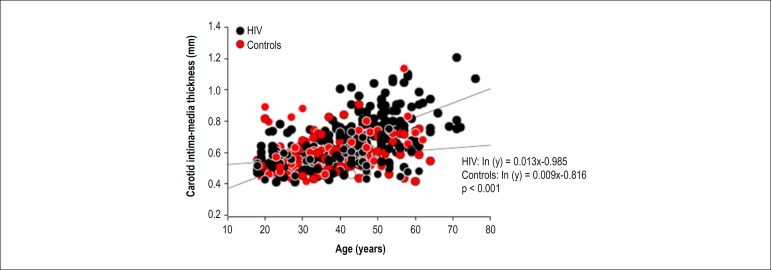
Association between carotid intima-media thickness and age in the control and HIV-infected group.

Figure 2 illustrates the significant association between age and CIMT in both groups, indicating that the oldest individuals have a higher CIMT, regardless of the presence of HIV infection. However, there was a significant interaction between age and the presence of HIV towards increasing CIMT (p < 0.001).

### Arterial stiffness

Comparing patients exposed and not exposed to PI, this drug class had no effect on arterial mechanical characteristics, expressed by PWV [7.10 (6.20-8.20) vs. 7.20 (6.30-8.40) m/s; p = 0.727] and AIx [28 (17-37) vs. 26 (13-38)%; p=0.315]. In addition, no effect was observed on CIMT [0.645 (0.570-0.765) vs. 0.625 (0.565-0.740) mm; p=0.331].

**Figure 3:**
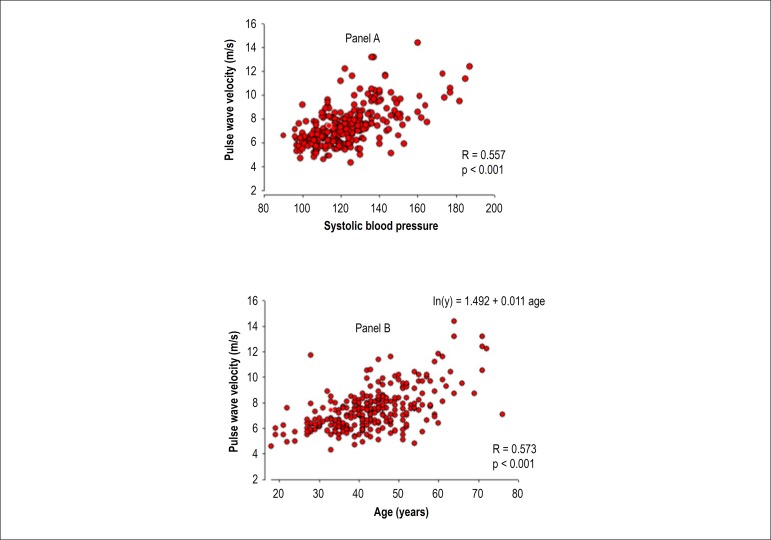
Association between pulse wave velocity and systolic blood pressure (panel A) and age (panel B).

 Pulse wave velocity was associated with age (R = 0.573, p < 0.001), CIMT (R = 0.449, p < 0.001) and SBP (R = 0.557, p < 0.001), as shown in Figure 3.

The association between PWV and age persisted in the model corrected for smoking. However, smoking interacted with age to increase PWV (p = 0.05). The AIx was also associated with age (R = 0.411, p < 0.001), CIMT (R = 0.274, p < 0.001), and SBP (R = 0.348, p < 0.001).

Arterial stiffness index was elevated in patients with PL compared to no-plaque individuals with PWV [7.90 (7.0-9.5) vs. 6.80 (6.10-8.0) m/s; p < 0.001] and AIx [37 (25-42) vs. 24 (12-35) %; p < 0.001]. Furthermore, PL patients showed median CIMT about 0.170 mm greater than patients without injury [0.770 (0.680-0.910) vs. 0.597 (0.550-0.690) mm; p = 0.003].

### Framingham coronary heart disease risk score

The FCRS was estimated in 252 HIV-infected patients. Of those, 207 were classified as low-risk (82.1%), 31 as intermediate-risk (12.3%), and 14 (5.56%) as high-risk for developing CVD within 10 years.

**Figure 4:**
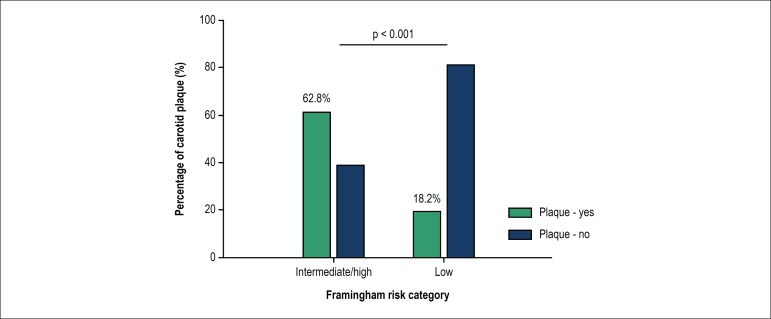
Frequency of plaques in HIV-positive patients according to risk stratification by Framingham risk score.

By grouping patients into two subgroups, low-risk (207 patients) and moderate/high-risk subgroups (45 patients), the PL frequency was 62.8% in the moderate/high-risk and 18.2% in the low-risk (p < 0.001) subgroup, as shown in Figure 4.

Low-risk individuals were 11 years younger than their moderate/high-risk counterparts (52.5 ± 10.3 years); p < 0.001.

Compared to the low-risk subgroup, the moderate/high-risk subgroup had higher CIMT [0.780 (0.710-0.935) vs. 0.605 (0.550-0.710) mm; p < 0.001], PWV [8.45 (7.15-10.05) vs. 6.90 (6.10-8.00) m/s; p < 0.001], TC [223 (188-253) vs. 182 (155-208) mg/dL; p < 0.001], LDL-c [130 (103-151) vs. 97.1 (79.6-126) mg/dL; p < 0.001], TGL [222 (160-309) vs. 143 (102-208) mg/dL; p < 0.001], fasting glucose [90 (80-102) vs. 83 (76-90) mg/dL; p = 0.002], and serum creatinine [0.90 (0.70-1.10) vs. 0.80 (0.70-0.90) mg/dL; p < 0.001], and lower HDL-c [38 (32-45) vs. 46 (37-56) mg/dL; p = 0.002].

From the 207 low-risk individuals, 83 had LDL-c lower than 130 mg/dL and were not using PI, and, of those, 14 (16.9%) were diagnosed with carotid artery PL (p = 0.036).

## Discussion

According to the Brazilian Ministry of Health data, the prevalence of overweight individuals in the general population is around 50%, while that of obesity is 12% to 17%.^33^ In the HIV-infected population, some studies have reported a prevalence of fat distribution changes of around 50%, with highly variable lipodystrophy data (20-80%) and obesity in 4-14%.^34,35^ In this study, BMI was lower in the HIV-group than in the control group (25.5 ± 4.5 vs. 27.4 ± 5.4 kg/m^2^). The overweight and obesity frequency was 51.1%, similar to that in the literature and lower than that found in the control group (63.1%).

The literature shows a higher frequency of smoking among HIV-infected individuals, reaching approximately 50%, than in the general population.^36,37^ We confirmed this in our series, finding active smoking in 43.6% of the patients and 16.1% of the control group (p < 0.001). Smoking had an effect on CIMT only in control subjects. This result could suggest that patients with HIV have other atherogenic factors that would neutralize the effects of smoking on intima-media thickness, with a tendency toward larger CIMT, independently of smoking.

LDL-c was associated with age (R = 0.252, p < 0.001) and time since HIV diagnosis (R = 0.293; p = 0.041), in direct association with increased serum levels.

These results suggest that atherosclerosis in the HIV population is influenced by other infection-related risk factors, in addition to presenting similar characteristics to the process classically described in other populations.^4-6,8,10,11^

Plaques were found in 37% of the HIV-infected individuals, slightly lower than the 55% reported in some publications.^18,19,37^

This study found that the presence of HIV produced an almost fivefold increase in the risk of carotid PL in the model adjusted for age, overweight/obesity, and smoking. We may therefore suppose that the presence of HIV infection is a contributory factor to the development of atherosclerosis, in addition to the traditional risk factors, and in agreement with other studies.^10,11,15,18,38^

There was no association between the presence of carotid PL and time since diagnosis or treatment duration, abdominal circumference, BMI, HDL-c, and CD4^+^ or CD8^+^ cell count. Other studies have reported similar results, suggesting that the increased risk of atherosclerosis in the presence of HIV is not directly associated with time since diagnosis, but with conditions involved in HIV infection.^10,38,39^

Patients with PL were 11 years older than those without PL, and predominantly male. Fasting glucose, TC, LDL-c, and TGL were significantly higher in patients with the diagnosis of PL. In patients with PL compared to patients without PL, SBP and DBP were 10 and 6 mm Hg higher, respectively. These results agree with those of other studies and strengthen the concept that atherogenesis in HIV-infected patients follows the classical risk factors described in other populations.^4,20,37,38^

Our results indicate that older individuals had higher CIMT, regardless of the presence of HIV infection; however, there was an interaction between age and the presence of HIV to increase CIMT (p < 0.001). Although time since diagnosis did not affect PL frequency, HIV infection appears to enhance the effect of age on CIMT. In this case, younger individuals with HIV could have vascular changes compatible with those of older patients. Understanding this behavior is important in screening for atherosclerosis in HIV-infected patients, because the protective effect of lower age would have less relevance.

In the HIV group, CIMT was associated with age (p < 0.001), BMI (p = 0.053), LDL-c (p = 0.005), and serum creatinine (p = 0.004); this was also seen in other studies.^17-19,37,40^ There were no associations with gender, smoking, diabetes, hypertension, statin therapy, HDL-c, or TGL. Interestingly, vessels may have PL with normal CIMT, which means that increased intima-media thickness and PL are not necessarily directly associated processes. However, both reflect the presence of endothelial dysfunction and are considered to favor cardiovascular events.^17-23^

Treatment with PI showed significant interaction with age and time since HIV diagnosis to increase TGL. Moreover, PI exposure was not associated with higher frequency of PL, in accordance with recent studies.^3,5,6,8,10,15,37-39^

In our study HAART showed correlation with unfavorable lipid profile, but without interfering in PL frequency or arterial stiffness.

The PWV was directly associated with age, CIMT, and SBP. These results are consistent with those from recent studies describing arterial stiffness indexes associated with age, hypertension, and vascular disease.^29-31^ In addition, AIx showed a direct association with age, CIMT, and SBP, with no interaction between age and smoking to increase AIx.

The elevation of PWV and AIx in patients with PL suggests that atherosclerosis is associated with functional alterations in the vessels; stiffer vessels have a higher risk of developing PL. Furthermore, patients with PL showed higher CIMT than patients without lesions, supporting the hypothesis that CIMT and atherosclerosis are associated, even when excluding the evolutionary nature of an alteration in the other.

This study included 207 patients classified as low risk (82.1%), 31 as moderate (12.3%) and 14 as high risk (5.56%) according to FCRS. The literature shows that the greater the FCRS, the greater the CIMT.^23^

In relation to age, younger patients were seen to have lower scores. This is in accordance with the concept that the FCRS, when applied to young individuals, can result in a low risk score, without implying that these individuals are not at risk of future cardiovascular events. It is important to note that almost 20% of the low-risk patients had carotid PL.

Patients in the moderate/high-risk subgroup showed unfavorable lipid profile with low HDL-c and elevated TC and LDL-c, as described in the literature, but no difference in hs-CRP, when compared to those classified as low risk by the FCRS.^12,15,25,26^ In addition, those classified as moderate/high-risk had higher intima-media thickness and PWV (p < 0.001), consistent with the hypothesis of a higher chance of vascular disease in the group.

Plaques were detected in 16% of the patients who were not treated with PI and had LDL-c lower than 130 mg/dL. This result, associated with the presence of PL in almost 20% of the low-risk patients classified by the FCRS, would indicate that they are at risk of developing atherosclerosis. Thus, the group with such characteristics would be exposed to major cardiovascular events, such as myocardial infarction and stroke, even without symptoms.

The IV Brazilian Guidelines on Dyslipidemia and Atherosclerosis Prevention recommend that evaluation of cardiovascular risk in HIV-infected patients should be performed assessing lipid profile and FCRS.^12^ Patients classified as low-risk have normal lipids and are not using HAART, and should undergo cardiovascular reevaluation in 2 years. In those with HAART, reevaluations are recommended one month after initiation of therapy and then every three months.

It is thus noted that the criteria established by the guidelines fail to consider the risks of this particular HIV-infected population, and that these patients have not been properly and specifically assessed for early CVD detection.

### Limitations

There are some limitations in this study. The data are only observational. There is a gap between this study population and the population from Framingham in the original description of the risk score. The pathophysiological determinants of multifactorial conditions involved in the association between HIV-infection or HAART use and atherosclerosis were not analyzed in this study. Information on previous CVD and other causes of immunosuppression were obtained by medical record review only, without specific evaluation for each condition.

## Conclusions

The data suggest that HIV-infected patients are at increased risk of atherosclerosis in association with the classical cardiovascular risk factors. In addition, HAART interacts with time since HIV infection diagnosis and patient age to modify lipid levels, but is not associated with higher PL frequency and does not promote functional changes in the arteries. Smoking, more prevalent in the HIV-infected population, influences the effect of age on the mechanical properties of arteries and may have an additional atherogenic effect in those patients. The FCRS may be inappropriate for this population.
